# Modeling Structure-Function Relationships in Synthetic DNA Sequences using Attribute Grammars

**DOI:** 10.1371/journal.pcbi.1000529

**Published:** 2009-10-09

**Authors:** Yizhi Cai, Matthew W. Lux, Laura Adam, Jean Peccoud

**Affiliations:** Virginia Bioinformatics Institute, Virginia Polytechnic Institute and State University, Blacksburg, Virginia, United States of America; University of Washington, United States of America

## Abstract

Recognizing that certain biological functions can be associated with specific DNA sequences has led various fields of biology to adopt the notion of the genetic part. This concept provides a finer level of granularity than the traditional notion of the gene. However, a method of formally relating how a set of parts relates to a function has not yet emerged. Synthetic biology both demands such a formalism and provides an ideal setting for testing hypotheses about relationships between DNA sequences and phenotypes beyond the gene-centric methods used in genetics. Attribute grammars are used in computer science to translate the text of a program source code into the computational operations it represents. By associating attributes with parts, modifying the value of these attributes using rules that describe the structure of DNA sequences, and using a multi-pass compilation process, it is possible to translate DNA sequences into molecular interaction network models. These capabilities are illustrated by simple example grammars expressing how gene expression rates are dependent upon single or multiple parts. The translation process is validated by systematically generating, translating, and simulating the phenotype of all the sequences in the design space generated by a small library of genetic parts. Attribute grammars represent a flexible framework connecting parts with models of biological function. They will be instrumental for building mathematical models of libraries of genetic constructs synthesized to characterize the function of genetic parts. This formalism is also expected to provide a solid foundation for the development of computer assisted design applications for synthetic biology.

## Author Summary

Deciphering the genetic code has been one of the major milestones in our understanding of how genetic information is stored in DNA sequences. However, only part of the genetic information is captured by the simple rules describing the correspondence between gene and proteins. The molecular mechanisms of gene expression are now understood well enough to recognize that DNA sequences are rich in functional blocks that do not code for proteins. It has proved difficult to express the function of these genetic parts in a computer readable format that could be used to predict the emerging behavior of DNA sequences combining multiple interacting parts. We are showing that methods used by computer scientists to develop programming languages can be applied to DNA sequences. They provide a framework to: 1) express the biological functions of genetic parts, 2) how these functions depend on the context in which the parts are placed, and 3) translate DNA sequences composed of multiple parts into a model predicting how the DNA sequence will behave in vivo. Our approach provides a formal representation of how the biological function of genetic parts can be used to assist in the engineering of synthetic DNA sequences by automatically generating models of the design for analysis.

## Introduction

“How much can a bear bear?” This riddle uses two homonyms of the word “bear”. The first instance of the word is a noun referring to an animal, and the second is a verb meaning “endure”. Although the word “bear” has over 50 different meanings in English, its meaning in any given sentence is rarely ambiguous. In a simple case like this riddle, the meaning of each word can be deciphered by looking at other words in the same sentence. In other cases, it is necessary to take into account a broader context to properly interpret the word. For instance, it may be necessary to read several sentences to decide if “bear claw” refers to a body part or a pastry. A reader will progressively derive the meaning of a text by recognizing structures consistent with the language grammar. It is often difficult to understand the meaning of a text by relying exclusively on a dictionary.

It is interesting to compare this bottom-up emergence of meaning with the top-down approach that made genetics so successful. The discipline was built upon a quest to define hereditary units that could be associated with observable traits well before the physical support of heredity was discovered [1],[2]. The one-to-one relationship between genes and traits was later refined by Beadle and Tatum's hypothesis that the gene action was mediated by enzymes [3],[4]. Cracking the genetic code has been one of the major milestones in understanding the information content of nucleic acids sequences. By demonstrating the colinearity of DNA, RNA, and protein sequences, the genetic code was instrumental in the identification of specific DNA sequences as genes. The influence of this legacy on contemporary biology cannot be underestimated. Models used in quantitative genetics predict phenotypes from unstructured lists of alleles at different loci [5],[6]. Similarly, genome annotations remain very gene-centric. Most bioinformatics databases have been designed to collect information relative to coding regions or candidate genes. Few, if any, annotations of non-coding regions or higher order structures are being systematically recorded even for model organisms like yeast [7],[8].

Yet, despite its success, the notion of gene appears insufficient to express the complexity of the relation between an organism genome and its phenotype [1],[9] The elucidation of the molecular mechanisms controlling gene expression has revealed a web of molecular interactions that have been modeled mathematically to show that important phenotypic traits are the emerging properties of a complex system [10]–[15]. The development of this more integrated understanding of the cell physiology leads to a progressive adoption of the more neutral notion of genetic part as a replacement for the notion of genes associated with specific traits. Making sense of the list of parts generated in genomics, proteomics, and metabolomics has been a major challenge for the systems biology community [16]–[21].

It is becoming apparent that the genetic code captures only a small fraction of the information content of DNA molecules [22],[23]. Yet, if there is a general agreement that the cell dynamics is somehow coded in genetic sequences, no formal relationship between DNA sequences and dynamical models of gene expression has been proposed so far. In particular, the formalization of the biological functions of genetic parts has remained elusive. As a result, building models of gene networks encoded in DNA sequences remains a labor-intensive process. This limitation has hampered the development of large families of models needed to analyze phenotypic data generated by libraries of related genetic constructs [24]–[28].

Synthetic biology is likely to be instrumental in refining our understanding of the design of natural biological systems [29]. Just like the genetic code was partly elucidated through the *de novo* chemical synthesis of DNA molecules [30],[31], the redesign of genomic sequences will shed a new light on the relations between structure and function in genetic sequences [32]–[34]. By considering biological parts as the building blocks of artificial DNA sequences [35], designing new parts that do not exist in nature [26]–[28], and making parts physically available to the community [36], synthetic biology calls for a systematic functional characterization of genetic parts [37]. These efforts are still limited by the difficulty in expressing how the function of biological parts may be influenced by the structure of the DNA sequence in which they are used. It has been shown that a partial redesign of the genomic sequences of two viruses had a significant effect on the virus fitness even though the redesigns preserved the protein sequences [33],[38]. Just as the context of the expression “bear claw” helps understand its meaning, it is necessary to consider the entire structure of the DNA molecule coding for particular genes to appreciate how those genes contribute to the phenotype.

One possible approach to this problem is to extend the linguistic metaphor used to formulate the central dogma. The notions of genetic code, transcription, and translation are derived from a linguistic representation of biological sequences. Several authors have modeled the structure of various types of biological sequences using syntactic models [39]–[46]. However, these structural models have not yet been complemented by formal semantic models expressing the sequence function. An interesting attempt to use grammars to model the dynamics of gene expression did not rely on a description of the DNA sequence structure. Instead, this grammar described how various inducible or repressible promoters can transition between different states under the control of environmental parameters [47]. The simple semantic model stored in a knowledge base established a correspondence between the strings generated by the syntax and the physiological state of the cell. The Sequence Ontology [48] and the Gene Regulation Ontology [49] represent other attempts to associate semantic values with biological sequences. Their controlled vocabularies can be used by software applications to manage knowledge. However, the semantics derived from these ontologies is a semantics of the sequence annotation, not of the sequences themselves.

## Model

We recently described a fairly simple syntactic model of synthetic DNA sequences [50] capable of generating a large number of previously published synthetic genetic constructs [24],[25],[51]. We have now enhanced this initial syntactic model with a formal semantic model capable of expressing the dynamics of the molecular mechanisms coded by the DNA sequences. Specialized terms like syntax, semantics, and others are defined in Table 1. Our approach uses attribute grammars [52], a theoretical framework developed in the 60s to establish a formal correspondence between the text of a computer program and the series of microprocessor operations it codes for [53],[54]. Even though other types of semantic models have been developed since then [55],[56], attribute grammars still represent a good compromise between simplicity and expressivity, an important characteristic to ensure that the framework can be used by non-computer scientists. Attribute grammars make it possible to use well characterized compilation algorithms to translate a DNA sequence into a mathematical model of the molecular interactions it codes for. As the static source code of a program directs the dynamic series of operations carried out by the microprocessor based on user inputs, the compilation process translates the static information of cells coded by DNA sequences into a dynamical model of the development of a phenotype in response to environmental influences [57].

**Table 1:** Glossary of specialized terms used throughout this article.

**Attribute grammar**	An attribute grammar is a context free grammar augmented with attributes, semantic rules, and conditions. Attribute grammars were developed as a means of formalizing the semantics of a context free grammar.
**Context free grammar**	A context free grammar is a quadruple (V, Σ, P, S) where V is a finite set of non-terminal symbols, Σ (the alphabet) is a finite set of terminal symbols, P is a finite set of rules, and S is a distinguished element of V called the start symbol. A rule P is of the following form A→ω where A is a single non-terminal symbol and ω is a string of terminals and/or non-terminals (possibly empty). The term “context-free” expresses the fact that non-terminals are rewritten without regard to the context in which they occur.
**Cusp bifurcation**	A codimension 2 bifurcation formed by the tangential meeting of two loci of saddle-node bifurcations. In other words, a cusp bifurcation traces the path of the points bounding a bistable region as they change with changes in two parameters. Bistability is implied within the cusp bounds.
**Direct left recursion**	A direct left recursion in context free grammar refers to rules of the form A→Aω. Parsing left recursion can possibly lead the parser down an infinite branch of the search tree in the corresponding logic program.
**PoPS**	The measurement of polymerase per second transcribing past a defined point of DNA.
**SBML**	The Systems Biology Markup Language (SBML) is a machine-readable language, based on XML, for representing models of biochemical reaction networks.
**Semantics**	Semantics reveals the meaning of syntactically valid strings in a language. For natural languages, this means correlating sentences and phrases with the objects, thoughts, and feelings of our experiences. For programming languages, semantics describes the behavior that a computer follows when executing a program in the language.
**Syntax**	Syntax refers to the ways symbols may be combined to create well-formed sentences (or programs) in a language. Syntax defines the formal relations between the constituents of a language, thereby providing a structural description of the various expressions that make up legal strings in the language. Syntax deals solely with the form and structure of symbols in a language without any consideration given to their meaning.

**Figure 1:**
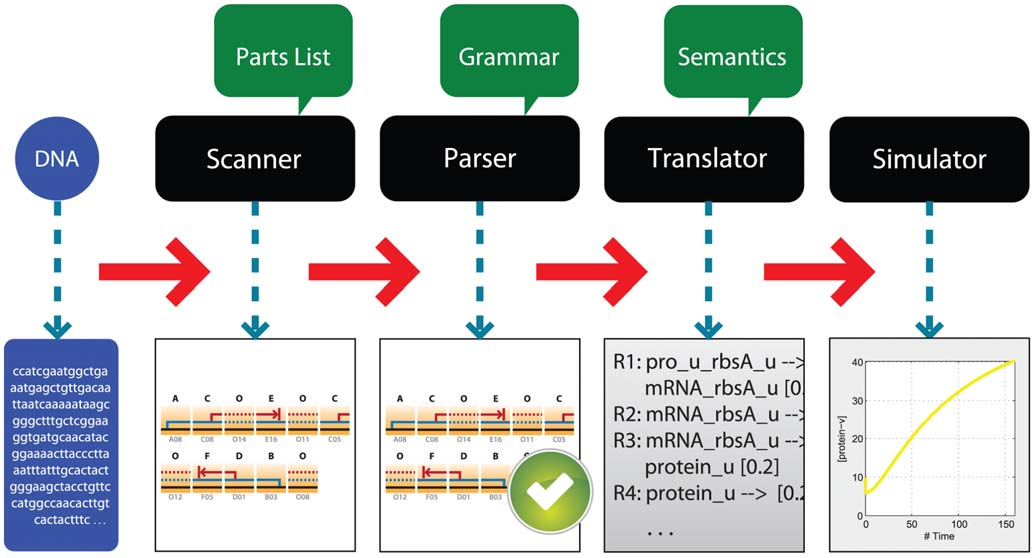
Workflow of generating the gene network model encoded in a DNA sequence. The input for this process is a DNA sequence that is first broken down into parts by the scanner. The combination of the parts is validated by the parser according to a syntactic model. After validation by the parser, the sequence is translated by applying semantic actions attached to the rules to transform the series of parts into a set of chemical equations. The resulting equations can then be solved using existing simulation engines. Each step takes the output of the previous step as input, so the workflow can start from any step if the appropriate input is provided.

**Figure 2:**
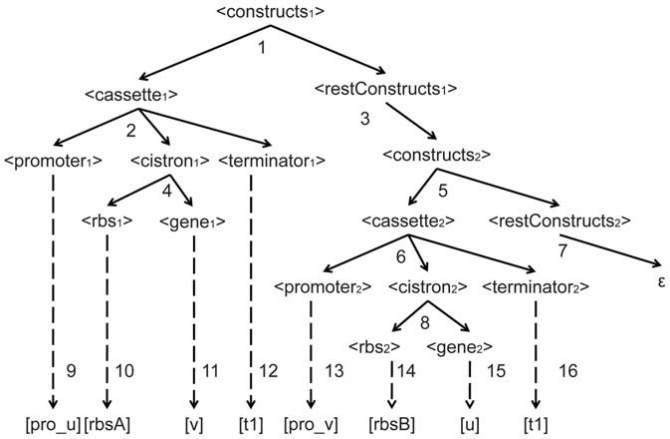
Parse tree showing the derivation process of a two-cassette genetic construct. In the derivation tree, terms in <> corresponds to the non-terminals in the grammar, while terms in [ ] are terminals, and the dashed lines indicate the transformation to terminals. The subscripts are used to distinguish different instances of the same category.

The translation of a gene network model from a genetic sequence is very similar to the compilation of the source code of a computer program into an object code that can be executed by a microprocessor (Figure 1). The first step consists in breaking down the DNA sequence into a series of genetic parts by a program called the lexer or scanner. Since the sequence of a part may be contained in the sequence of another part, the lexer is capable of backtracking to generate all the possible interpretations of the input DNA sequences as a series of parts. All possible combinations of parts generated by the lexer are sent to a second program called the parser to analyze if they are structurally consistent with the language syntax. The structure of a valid series of parts is represented by a parse tree [50] (Figure 2). The semantic evaluation takes advantage of the parse tree to translate the DNA sequence into a different representation such as a chemical reaction network. The translation process requires attributes and semantic actions. Attributes are properties of individual genetic parts or combinations of parts. Semantic actions are associated with the grammar production rules. They specify how attributes are computed. Specifically, the translation process relies on the semantic actions associated with parse tree nodes to synthesize the attributes of the construct from the attributes of its child nodes, or to inherit the attributes from its parental node. In our implementation, the product of the translation is a mass action model of the network of molecular interactions encoded in the DNA sequence. By using the standardized format of Systems Biology Markup Language (SBML), the model can be analyzed using existing simulation engines [58]–[60].

## Results

### Compilation of a DNA sequence

**Figure 3:**
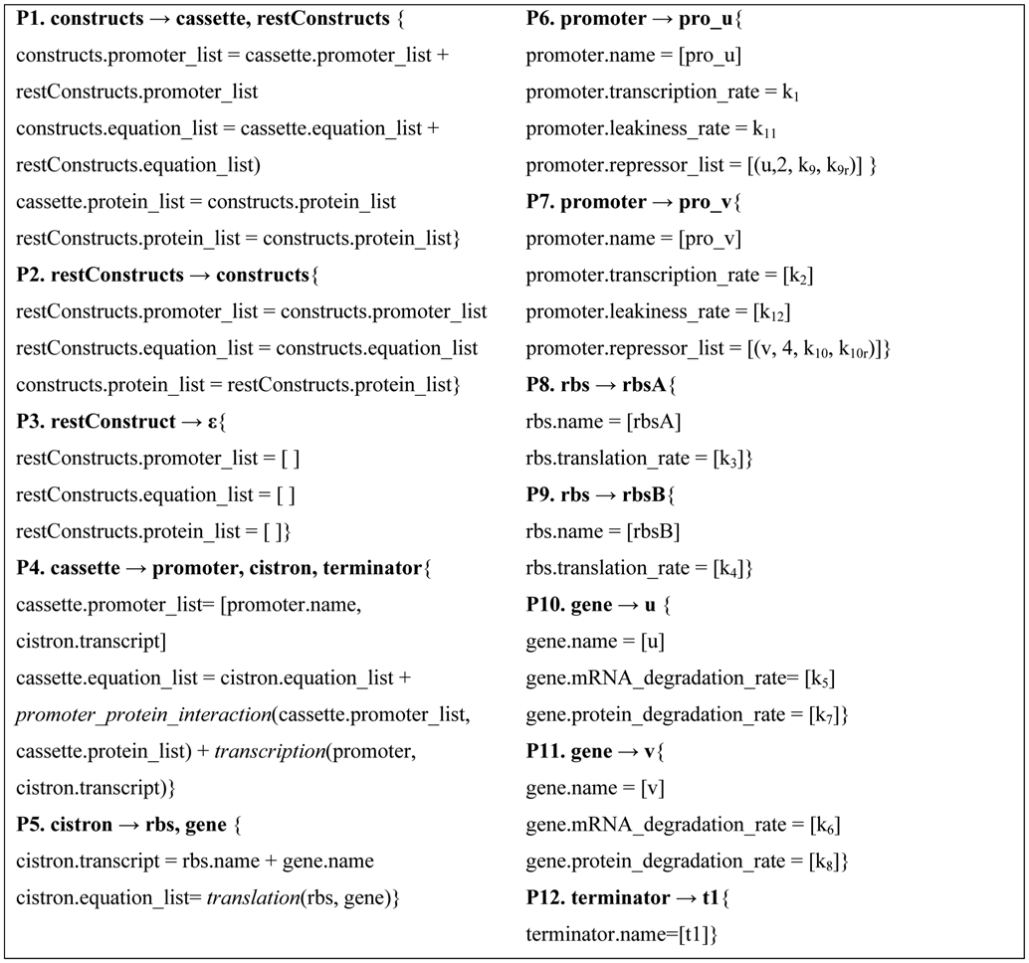
An example of attribute grammar.

We have developed a simple grammar compact enough to be presented extensively, yet sufficiently complex to represent basic epistatic interactions. The grammar generates constructs composed of one or more gene expression cassettes. The gene expression cassettes are themselves composed of a promoter, cistron, and transcription terminator. Finally, a cistron is composed of a Ribosome Binding Site (RBS) and a coding sequence (gene). The syntax is composed of 12 production rules (P1 to P12) displayed in bold characters in Figure 3 where each entry is composed of a rewriting rule (bold), and semantic actions (curly brackets). The symbol ε refers to an empty string, [ , ] to a list, [] to an empty list, and the ‘+’ sign indicates the concatenation operation on two lists. This syntax is comparable to the one described previously [50] except that we introduced the extra non-terminal restConstructs to allow the generation of constructs with multiple cassettes without introducing parsing problems due to direct left recursions [61].

The attributes of a part include the kinetic rates related to this part and the interaction information. For example, the attributes of a promoter include a transcription rate along with a list of proteins repressing it and the kinetic parameters of the protein-DNA interactions. For non-terminal variables corresponding to combinations of parts such as cistrons, the attributes include a list of proteins, a list of promoters, and a list of chemical equations. The equation list is used to store the model of the system behavior, while the lists of promoters and proteins are recorded for computing the molecular interactions resulting from the DNA sequence. The complete set of attributes used in this simple grammar is listed in Table 2.

**Table 2:** Attributes associated with non-terminals.

Non-terminals	Inherited Attribute	Synthesized Attributes
constructs	protein_list	promoter_list, equation_list
cassette	protein_list	promoter_list, equation_list
restConstructs	protein_list	promoter_list, equation_list
cistron	protein_list	transcript, equation_list
promoter	-	name, transcription_rate, leakiness_rate, repressor_list
RBS	-	name, translation_rate
gene	-	name, mRNA_degradation_rate, protein_degradation_rate
terminator	-	name

**Figure 4:**
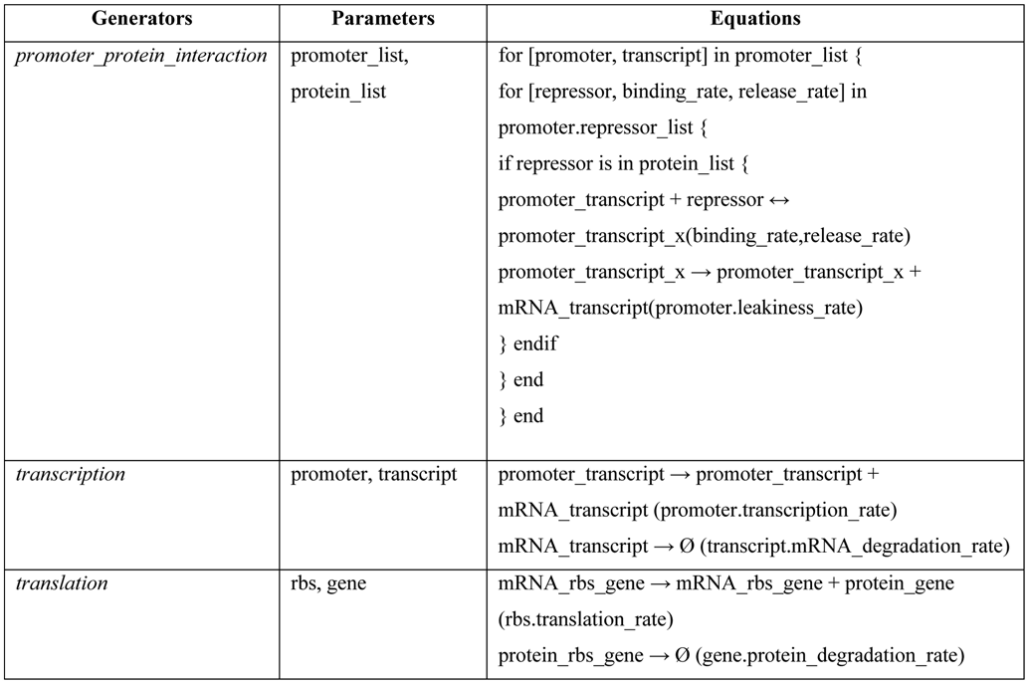
Equation generators.

If many attributes can be computed locally by only considering a small fragment of the DNA sequence, other attributes are global properties of the system. For instance, the computation of protein-DNA interactions requires access to a global list of proteins expressed by the constructs. However, this list is not available until all of the different cassettes have been parsed. The problem is overcome by using a multiple-pass compilation method. In the first pass, the compiler does not do any structural validation but builds the list of proteins in the system and passes the list as an inherited attribute to the second pass. In the second pass, the promoter-protein interactions can be calculated locally at the level of each cassette. Rules P1 to P5 define the structure of a design, while rules P6 to P12 cover the selection of a specific part for each category. In the semantic action, the relation between an attribute and its variable is indicated by a dot and constants are enclosed by brackets. For instance, gene.mRNA_degration_rate = [k_6_] indicates that the value of the attribute mRNA_degration_rate of a gene is a constant k_6_. The attribute repressor_list used in P6 and P7 includes the name of the repressor, the stoichiometry, and the kinetic constants of the forward and reverse reactions of the protein-DNA interaction. Table S1 details the parsing steps and computational dependence of each step. Finally, the equation writing operations are handled by functions typed in italics in Figure 3 and defined in Figure 4.

**Figure 5:**
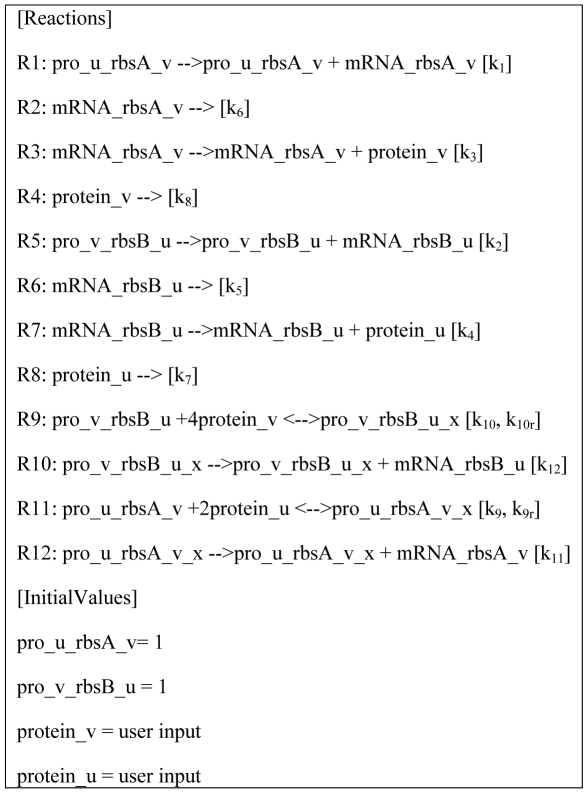
Chemical equations translated from a DNA sequence.

The translation of the DNA sequence into a mathematical model is available as the equation_list attribute of constructs. The model outputs are generated by equations generators, which are purposely decoupled from the semantic actions. The decoupling enables the flexibility of using different equation formats to describe a biological process. The translation of the construct composed of the parts pro_u rbsA gene_v t1 pro_v rbsB gene_u t1 generates the equations displayed in the [Reactions] section of Figure 5. Each line is composed of a reaction index (R1 to R12), the chemical equation itself, and one or two reaction parameters depending on the reaction reversibility. The initial values have been computed by assigning 1 to variables representing DNA sequences and prompting the user to set the initial condition of proteins. The scripts and data used in this report are available in Dataset S1.

### Expressing context-dependencies of parts function

The semantic model presented in the previous section is completely modular since the parameters of the model describing the construct behavior are attributes of individual parts, not of higher order structures. For instance, in the previous model (Figures 3 and 4), translational efficiency is primarily determined by the RBS sequence [62],[63]. This association between RBS and translation rate was successfully used to design one of the first artificial gene networks [24] and is still used by many synthetic biology software applications [64]–[67]. Yet, it is also well known that translation initiation can be attenuated by stable mRNA secondary structures [68]–[70]. This leads to a situation where a translational rate can no longer be considered the attribute of an individual part but needs to be considered as the attribute of a specific combination of parts. This type of context-dependency can naturally be expressed using attribute grammars since the translation reaction is computed at the cistron level, not at the level of individual parts. Rule P5 of Figure 3 can be modified by introducing a new function to retrieve the translation rate for specific combination of gene and RBS.

P5. cistron → rbs, gene

{

cistron.translation_rate = get_translation_rate (rbs, gene)

cistron.transcript = rbs.name+gene.name

cistron.equation_list = translation(rbs, gene, cistron.translation_rate)

}

The get_translation_rate function checks for specific cases of interactions between an RBS and coding sequence first. If none is found, then the default RBS translation rate is used.

If exists translation_rate(rbs, gene)

translation_rate = translation_rate(rbs, gene)

else

translation_rate = translation_rate(rbs)

endif

This approach is illustrated in Table 3 using previously published data demonstrating the interference between the RBS and coding sequence [68]. Specifically, this report provides the relation expression observed in 23 different constructs generated by combining different variants of the RBS and MS2 coat protein gene. This data set has been reorganized in Table 3 by sorting the constructs according to the RBS and gene variants they used. Three of the constructs using the WT RBS sequence resulted in a maximum level of expression while the expression of the gene variants ORF4, ORF5, and ORF6 were expressed at a much lower level due to the greater stability of the mRNA secondary structure. A similar pattern is observed for other RBS variants (RBS1, RBS2, RBS3, RBS7). For all of these RBS variants, it is possible to define the translation_rate function by associating the default translation rate with the maximum expression rate. Specific translation rates associated with particular pairs of RBS and gene variants are recorded separately.

**Table 3:** Context-dependency of experimentally determined translation rates.

Mutant	RBS	ORF	Expression	Translation rate function
1	RBS WT	ORF WT	100	translation_rate(RBS WT)
6	RBS WT	ORF2	100	translation_rate(RBS WT)
7	RBS WT	ORF3	100	translation_rate(RBS WT)
17	RBS WT	ORF4	3	translation_rate(RBS WT, ORF4)
20	RBS WT	ORF5	6	translation_rate(RBS WT, ORF5)
23	RBS WT	ORF6	0.3	translation_rate(RBS WT, ORF6)
4	RBS1	ORF WT	100	translation_rate(RBS1)
2	RBS1	ORF1	100	translation_rate(RBS1)
3	RBS1	ORF2	100	translation_rate(RBS1)
5	RBS1	ORF3	4	translation_rate(RBS1, ORF3)
14	RBS1	ORF4	<0.003	translation_rate(RBS1, ORF4)
9	RBS2	ORF WT	100	translation_rate(RBS2)
8	RBS2	ORF1	100	translation_rate(RBS2)
10	RBS2	ORF3	100	translation_rate(RBS2)
12	RBS3	ORF WT	100	translation_rate(RBS3)
11	RBS3	ORF1	20	translation_rate(RBS3, ORF1)
13	RBS3	ORF3	100	translation_rate(RBS3)
15	RBS4	ORF4	0.1	translation_rate(RBS4)
16	RBS5	ORF4	0.05	translation_rate(RBS5)
22	RBS6	ORF WT	0.2	translation_rate(RBS6, ORF WT)
18	RBS6	ORF4	80	translation_rate(RBS6)
21	RBS7	ORF WT	100	translation_rate(RBS7)
19	RBS7	ORF4	100	translation_rate(RBS7)

### Exploration of genetic design space

**Figure 6:**
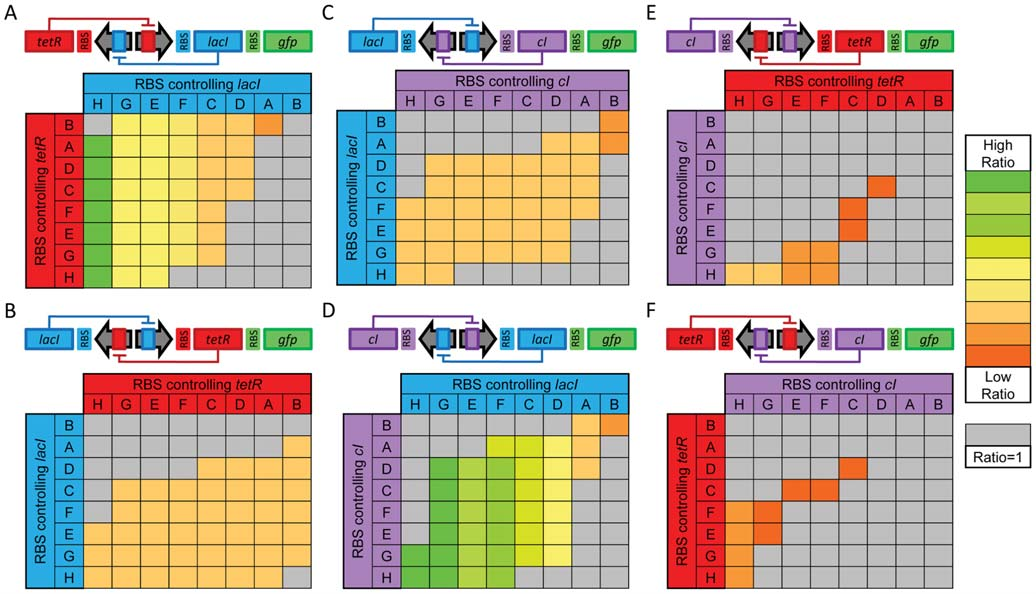
Mapping the behavior of 384 genetic constructs. Each section A to F indicates a different selection of repressors within a toggle switch: (A) *tetR* and *lacI*, (B) *lacI* and *tetR*, (C) *lacI* and *cI*, (D) *cI* and *lacI*, (E) *cI* and *tetR*, and (F) *tetR* and *cI*. Other networks that cannot give rise to bistability (e.g. a construct with *tetR* as both genes) are excluded as are designs that only vary the GFP RBS (see text). Each pair is explored by varying the RBS (ordered by translational efficiency from low (RBS H) to high (RBS B) as determined by a qualitative fit of the results of Gardner et al. [24] with consistent letter-based labels) and calculating the detectability ratio, defined as the steady state GFP concentration in the “on” state divided by the concentration in the “off” state. These ratios are displayed using a color map as indicated by the legend to the right. Monostable constructs have a ratio of 1 and are indicated by gray boxes. The ratio gives a measure of how easily the two steady states can be distinguished, which is important due to high experimental noise. Each pane also elucidates the traditional two-parameter bifurcation diagram of each gene pair as the translational rates are varied by changing RBSs. Constructs near the edge of the cusp operate near saddle-node bifurcations and are more prone to noise-induced switching. Thus, constructs from the cusp interior are preferred for robust behavior.

The semantic model in Figures 3 and 4 is a compact proof of concept example, but it does not capture a number of features commonly found in actual genetic constructs. In order to demonstrate that our approach is capable of modeling more realistic DNA sequences, we have extended this semantic model (Supplementary Materials) to translate the DNA sequences of previously published DNA plasmids that include polycistronic cassettes in different orientations [24]. This plasmid library was generated by 32 different genetic parts (three promoters: p_L_tetO-1, p_L_s1con, ptrc-2; eight RBS: rbsA to rbsH; and four genes: *tetR*, *cIts*, *lacI*, and *gfp* and one terminator, all in both orientations). The syntax generates 72 different single gene expression constructs in each orientation. By combining two genes repressing each other in a construct, it is possible to make bistable artificial gene networks that are represented in Figure 6. These bistable networks can be used as a genetic switch.

To demonstrate the potential use of a semantic model to search for a desirable behavior in a large genetic design space, we have generated the DNA sequences of all 41,472 possible sequences (72^2^×8 RBS for the reporter gene) having the same structure as previously described switches. All sequences were translated into separate model files and a script was developed to perform a bistability analysis of each model. Parameters of the semantic model were obtained by qualitatively matching the experimental results of the six previously published switches [24] and are summarized in Table S2. Most of the automatically generated sequences led to inherently non-bistable networks because the necessary repressor/promoter pairs did not match. Since this specific example is particularly well understood, we could have generated a limited number of targeted constructs. Yet, we chose to generate all possible sequences to demonstrate the generality of our approach. In particular, it was important to evaluate the computational cost of generating and translating DNA sequences to ensure that it would not prevent a systematic exploration of more complex design spaces. It takes only minutes to generate 41,472 sequences and translate them into SBML files. Hence, the computational cost of this step is negligible compared to the time required by the simulation of the SBML files.

Bistability was tested numerically by integrating the differential equations until they converged to a steady state starting from two different initial conditions. The two initial conditions started with one protein level very high and the other very low and vice versa. We characterized the bistability by computing the ratio of reporter concentration for the two steady state values. In order to globally verify the behavior of this large population of models, we focused on the 3,072 constructs potentially capable of bistability, 1,408 of which were found to be bistable. We further reduced the number of constructs used to verify the translation process from 3,072 to 384 by assuming that two constructs differing only in the RBS in 5′ of the reporter gene would produce the same ratio of steady state values. Figure 6 visualizes the behavior of these 384 constructs. Constructs that are not bistable have a ratio of 1. This ratio gives insight into how the construct is expected to be experimentally detectable. Since most experimental methods cannot give an exact value of protein concentration, a high ratio is desired to rise above experimental noise. Each of the 6 windows is analogous to the previously described two-parameter bifurcation diagram for that pair of repressors [24]. This gives confidence that both the semantic model of DNA sequences and the compiler used to translate automatically generated DNA sequences give results consistent with manually developed models of this family of gene networks. In the long term, the advantage to our approach over a traditional two-parameter bifurcation is the association of discrete parameter values with specific parts. This will prove particularly valuable when the context-dependencies of parameter values are better documented experimentally.

This example demonstrates the benefit of building a semantic model of synthetic DNA sequences. Even a small library of genetic parts can generate large numbers of artificial gene networks having no more than a few interacting genes. A syntactic model describing how parts can be combined into constructs is a compact representation of the genetic design space generated from the parts library. While it is possible to manually build mathematical models capturing the dynamics of some of these artificial gene networks individually, it becomes desirable to automate the process to ensure the model consistency when building large families of related models derived from the same parts library. By considering genetic parts as the terminal symbols of an attribute grammar, it becomes possible to automatically generate models of numerous artificial gene networks derived from this parts library and quickly identify the optimal designs [71].

## Discussion

### Computer assisted design of synthetic genetic constructs

The parameter values used in the previous example were selected to match an extremely small set of six experimental data points. Although the under-determination of the model does not make it possible to precisely estimate the value of these parameters, the example illustrates how the framework could provide valuable guidance in selecting specific parts for a design. Considering that the exact value of parameters for parts is still a far off perspective, the automatic exploration of the design space presented here will provide useful guidance in construct design. For example, robust constructs from the cusp interior of the *tetR*/*cI* and *lacI*/*cI* pairings could be built and tested while less robust switches based on the *lacI*/*tetR* pairing would be avoided. As more is learned about these parts including the specific rates in different genetic contexts, the predictive ability of such maps will increase. Other motifs could be explored in a similar manner. For example, oscillators [11] could be explored by permuting parts and calculating the model-predicted existence of oscillations as well as their period or amplitude.

The approach presented in this report will be implemented into GenoCAD [72], the web-based tool we have developed to give biologists access to our syntactic design framework. Through GenoCAD, users will benefit from the syntactic and semantic models of various parts sources (GenoCAD provided library, MIT Registry of Standard Biological Parts, or user created parts library). Initially, users will be able to translate their designs into SBML files that could be imported in SBML-compliant simulation tools (www.sbml.org/SBML_Software_Guide) for further analysis. At a later stage, simulation results and more advanced numerical analyses will be seamlessly integrated in GenoCAD's workflow. One of the major obstacles toward the implementation of such semantic models in GenoCAD is the development of a data model allowing users to understand and possibly edit the functional model of the parts they use.

A function description language called Genetic Engineering of living Cells (GEC) was recently introduced to specify the properties of a design [67]. GEC is capable of finding a DNA sequence that implements the desirable phenotypic functions. Several other software applications have been recently released to design biological systems from standardized genetic parts. ASMPART [65], SynBioSS [66], a specialized ProMot package [64] and TinkerCell (www.tinkercell.com) illustrate this trend. These tools are still exploratory. One of their limitations is the requirement to define parts in a specialized format, such as SBML or Modeling Description Language (MDL). Furthermore, instead of defining parts interactions in the underlying parts data models, these tools rely on the user to manually define them textually [66] or graphically [64]. As a result of this specific limitation, several of these tools do not appear suitable for the automatic exploration of a design space. Moreover, they tend to rely on a loosely defined relationship between the structure of the genetic constructs and their behavior. They allow parts to be assembled in any order without regard for biological viability.

Still, the scripts developed to generate our results are of lesser importance than the application of the theory of semantics-based translation using attribute grammars to the translation of DNA sequences into dynamical models representing the molecular interactions they encode. Since this approach is used to develop the compilers of many computer languages [56],[73], a wealth of existing theoretical results and software tools can find new applications in the life sciences. For instance, we have implemented semantic models of DNA sequences into two widely used but very different programming environments, Prolog [74] and ANTLR [75]. Future research efforts will need to investigate the pros and cons of different compiler generators and different parsing algorithms for analyzing even genome-scale DNA sequences and how they impact the ability of grammars to express various features of DNA sequences. Also, the type of attributes associated with parts is flexible. Here we primarily use mass action kinetic rates as attributes, but we could just as easily have used an emerging synthetic biology unit like polymerase per second (PoPS) [37],[76].

Ultimately, tools capable of automatically generating models of the behavior of synthetic DNA sequences will be important for the advancement of synthetic biology [71]. However, these tools will need to be able to express that the contribution of a genetic part to the phenotype of an organism depends largely on the local and global context in which it is placed. The interference between RBS and coding sequence is just one example of the biological complexity that computer assisted design applications will have to properly consider.

### Functional characterization of genetic parts

Before it will be used to build synthetic genetic systems meeting user-defined specifications, the semantic model of DNA sequences presented in this report will be instrumental in the quantitative characterization of structure-function relationships in synthetic DNA sequences. The vision of applying quantitative engineering methods to biological problems has been recognized as a promising avenue to biological discovery [29]. The critical role of artificial gene networks in the characterization of molecular noise affecting the dynamics of gene networks [77] illustrates the potential of synthetic biology as a route to refine the understanding of basic biological processes.

Ongoing efforts aim to carefully define how parts should fit together syntactically and what attributes are needed to characterize their function. For example, the sequence between the RBS and the start codon has been shown to play an important role in translation rate [63]. The question arises whether the RBS should be defined to include the spacing, or if there should be a separate parts category for the spacer. The rapid development of gene synthesis techniques [78] will make it possible to investigate these questions with a base-level resolution. Beyond libraries of parts for designing expression vectors, similar curation efforts could lead to the identification of parts in genomic sequences, whereby the hypothetical function of these parts as they are expressed in attribute grammars could be tested by genome refactoring [33].

## Supporting Information

Table S1 Computation dependence corresponding to the derivation tree in Fig. 2 The computation starts from the leaves of the tree, and the semantic values computed are transferred to upstream nodes. The computation of each node cannot proceed until all of its sub-trees are computed. For example, the computation of semantic values of <constructs1> (2) is pending until its subtrees<cassette1> (3) and <restConstructs1> (4) are computed.
(0.01 MB PDF)

Table S2 List of parts used in the “exploration of genetic space” section and values of associated attributes
(0.01 MB PDF)

Dataset S1 Zip file containing the scripts and data used in this report.
(0.03 MB ZIP)
