# Supplementary material for: Modeling Structure-Function Relationships in Synthetic DNA Sequences using Attribute Grammars
Source: PLoS Comput Biol. 2009 Oct 9;5(10):e1000529. doi: 10.1371/journal.pcbi.1000529 (PMC2748682; doi:10.1371/journal.pcbi.1000529)
Supplement: Table S1 — Computation dependence corresponding to the derivation tree in Fig. 2 The computation starts from the leaves of the tree, and the semantic values computed are transferred to upstream nodes. The computation of each node cannot proceed until all of its sub-trees are computed. For example, the computation of semantic values of (2) is pending until its subtrees (3) and (4) are computed. (0.01 MB PDF) [file pcbi.1000529.s001.pdf]

No.	Semantic Actions	Dependence
9	promoter1.name = [pro_u]	N/A
10	rbs1.name = [rbsA]	N/A
11	gene1.name = [v]	N/A
12	terminator1.name = [t1]	N/A
13	promoter2.name = [pro_v]	N/A
14	rbs2.name = [rbsB]	N/A
15	gene2.name = [u]	N/A
16	terminator2.name = [t1]	N/A
4	cistron1.transcript = rbs1.name + gene1.name = [rbsA_v]<br>cistron1.equation_list = *translation*(rbsA, v)	10, 11
8	cistron2.transcript = rbs2.name + gene2.name = [rbsB_u]<br>cistron2.equation_list = *translation*(rbsB, u)	14, 15
7	restConstruct2.equation_list = [ ]	N/A
2	cassette1.promoter_list = [promoter.name, cistron.transcript] = [pro_u, rbsA_v]<br>cassette1.equation_list = *promoter_protein_interaction*(cassette1.promoter_list, cassette1.protein_list) + *transcription*(promoter, cistron1.transcript) + cistron1.equation_list = *promoter_protein_interaction*([pro_u, rbsA_v], cassette1.protein_list) + *transcription*(pro_u, rbsA_v) + *translation*(rbsA, v)	4, 9, 12
6	cassette2.promoter_list = [promoter.name, cistron.transcript] = [pro_v, rbsB_u]<br>cassette2.equation_list = *promoter_protein_interaction*(cassette2.promoter_list, cassette2.protein_list) + *transcription*(promoter, cistron2.transcript) + cistron2.equation_list = *promoter_protein_interaction*([pro_v, rbsB_u], cassette2.protein_list) + *transcription*(pro_v, rbsB_u) + *translation*(rbsB, u)	8, 13, 16
5	construct2.equation_list = cassette2.equation_list + restConstructs2.equation_list = *translation*(rbsB, u) + *transcription*(pro_v, rbsB_u) + *promoter_protein_interaction*([pro_v, rbsB_u], cassette2.protein_list)	6, 7
3	restConstructs1.equations_list = construct2.equation_list = *translation*(rbsB, u) + *transcription*(pro_v, rbsB_u) + *promoter_protein_interaction*([pro_v, rbsB_u], cassette2.protein_list)	5
1	cassette1.protein_list = constructs1.protein_list = [protein_u, protein_v]<br>cassette2.protein_list = constructs1.protein_list = [protein_u, protein_v]<br>constructs1.equation_list = cassette1.equation_list + restConstructs.equation_list = *transcription*(pro_u, rbsA_v) + *translation*(rbsA, v) + *transcription*(pro_v, rbsB_u) + *translation*(rbsB, u)+ *promoter_protein_interaction*([pro_u, rbsA_v], [protein_u, protein_v]) + *promoter_protein_interaction*([pro_v, rbsB_u], [protein_u, protein_v])	2, 3

Table S1: Computation dependence corresponding to the derivation tree in Fig. 2. The computation starts from the leaves of the tree, and the semantic values computed are transferred to upstream nodes. The computation of each node cannot proceed until all of its sub-trees are computed. For example, the computation of semantic values of <constructs1> (2) is pending until its subtrees<cassette1> (3) and <restConstructs1> (4) are computed. The '+' symbol indicated the concatenation operation for two lists.
