# Supplementary material for: Modeling Structure-Function Relationships in Synthetic DNA Sequences using Attribute Grammars
Source: PLoS Comput Biol. 2009 Oct 9;5(10):e1000529. doi: 10.1371/journal.pcbi.1000529 (PMC2748682; doi:10.1371/journal.pcbi.1000529)
Supplement: Table S2 — List of parts used in the “exploration of genetic space” section and values of associated attributes (0.01 MB PDF) [file pcbi.1000529.s002.pdf]

Part Name	Part Type	Associated Attribute	Attribute Value
ptrc2	Promoter	promoter.name	ptrc2
		promoter.transcription_rate	25
		promoter.leakiness_rate	.25
		promoter.repressor_list	[[lacI, 4, 0.001, 1], [lacIrc, 4, 0.001, 1]]
pls1con	Promoter	promoter.name	pls1con
		promoter.transcription_rate	50
		promoter.leakiness_rate	0.00833333
		promoter.repressor_list	[[cIts, 2, 0. 1, 1], [cItsrc, 2, 0.1, 1]]
pltet01	Promoter	promoter.name	tetR
		promoter.transcription_rate	10
		promoter.leakiness_rate	0.1
		promoter.repressor_list	[[tetR, 2, 0.1, 1], [tetRrc, 2, 0. 1, 1]]
ptrc2rc	Reverse Promoter	reversePromoter.name	ptrc2rc
		reversePromoter.transcription_rate	25
		reversePromoter.leakiness_rate	.25
		reversePromoter.repressor_list	[[lacI, 4, 0.001, 1], [lacIrc, 4, 0.001, 1]]
pls1conrc	Reverse Promoter	reversePromoter.name	pls1conrc
		reversePromoter.transcription_rate	50
		reversePromoter.leakiness_rate	0.00833333
		reversePromoter.repressor_list	[[cIts, 2, 0. 1, 1], [cItsrc, 2, 0.1, 1]]
pltet01rc	Reverse Promoter	reversePromoter.name	tetRrc
		reversePromoter.transcription_rate	10
		reversePromoter.leakiness_rate	0.1
		reversePromoter.repressor_list	[[tetR, 2, 0.1, 1], [tetRrc, 2, 0. 1, 1]]
rbsA	RBS	rbs.name	rbsA
		rbs.translation_rate	25
rbsB	RBS	rbs.name	rbsB
		rbs.translation_rate	50
rbsC	RBS	rbs.name	rbsC
		rbs.translation_rate	10
rbsD	RBS	rbs.name	rbsD
		rbs.translation_rate	12.5
rbsE	RBS	rbs.name	rbsE
		rbs.translation_rate	6.25
rbsF	RBS	rbs.name	rbsF
		rbs.translation_rate	7
rbsG	RBS	rbs.name	rbsG
		rbs.translation_rate	5
rbsH	RBS	rbs.name	rbsH
		rbs.translation_rate	2
rbsArc	Reverse RBS	reverseRBS.name	rbsA
		reverseRBS.translation_rate	25
rbsBrc	Reverse RBS	reverseRBS.name	rbsB
		reverseRBS.translation_rate	50
rbsCrc	Reverse RBS	reverseRBS.name	rbsC
		reverseRBS.translation_rate	10
rbsDrc	Reverse RBS	reverseRBS.name	rbsD
		reverseRBS.translation_rate	12.5
rbsErc	Reverse RBS	reverseRBS.name	rbsE
		reverseRBS.translation_rate	6.25

rbsFrc	Reverse RBS	reverseRBS.name	rbsF
		reverseRBS.translation_rate	7
rbsGrc	Reverse RBS	reverseRBS.name	rbsG
		reverseRBS.translation_rate	5
rbsHrc	Reverse RBS	reverseRBS.name	rbsH
		reverseRBS.translation_rate	2
lacI	Gene	gene.name	lacI
		gene.mRNA_degradation_rate	1
		gene.protein_degradation_rate	0.1
gfpmut3	Gene	gene.name	gfpmut3
		gene.mRNA_degradation_rate	1
		gene.protein_degradation_rate	0.1
cIts	Gene	gene.name	cIts
		gene.mRNA_degradation_rate	1
		gene.protein_degradation_rate	0.1
tetR	Gene	gene.name	tetR
		gene.mRNA_degradation_rate	1
		gene.protein_degradation_rate	0.1
lacIrc	Reverse Gene	reverseGene.name	lacIrc
		reverseGene.mRNA_degradation_rate	1
		reverseGene.protein_degradation_rate	0.1
gfpmut3rc	Reverse Gene	reverseGene.name	gfpmut3rc
		reverseGene.mRNA_degradation_rate	1
		reverseGene.protein_degradation_rate	0.1
cItsrc	Reverse Gene	reverseGene.name	cItsrc
		reverseGene.mRNA_degradation_rate	1
		reverseGene.protein_degradation_rate	0.1
tetRrc	Reverse Gene	reverseGene.name	tetRrc
		reverseGene.mRNA_degradation_rate	1
		reverseGene.protein_degradation_rate	0.1
b0010	Terminator	none	
b0012	Terminator	none	
b0016	Terminator	none	
b0010rc	Reverse Terminator	none	
b0012rc	Reverse Terminator	none	
b0016rc	Reverse Terminator	none	

Table S2: List of parts used in the "exploration of genetic space" section and values of associated attributes.
